# Single nucleotide polymorphisms in tinnitus patients exhibiting severe distress

**DOI:** 10.1038/s41598-020-69467-0

**Published:** 2020-08-03

**Authors:** Takahisa Watabe, Sho Kanzaki, Noriko Sato, Tatsuo Matsunaga, Masaaki Muramatsu, Kaoru Ogawa

**Affiliations:** 1 Department of Otolaryngology, Keio University School of Medicine, Tokyo, Japan; 2 Molecular Epidemiology, Medical Research Institute Tokyo Medical and Dental University, Tokyo, Japan; 3 Division of Hearing and Balance Research, National Institute of Sensory Organs, National Hospital Organization Tokyo Medical Center, Tokyo, Japan

**Keywords:** Medical research, Neurology

## Abstract

The association between distress caused by tinnitus and psychological factors such as depression and anxiety has been examined and reported. However, prognostic factors remain poorly understood because there are only a few reports on genetic associations. We theorized there might be an association between the grade of tinnitus distress and the genetic background related to psychological factors which might lead us to identify prognostic markers. We enrolled 138 patients who had suffered from tinnitus for over 3 months. Using Tinnitus Handicap Inventory (THI) scores, we examined the association between tinnitus distress and a genetic background related to depression or anxiety. A significant association between single nucleotide polymorphism rs131702 of the Breakpoint Cluster Region (*BCR*) gene and the severe THI score was identified. In addition, there was an association with the severity of the State-Trait Anxiety Inventory, an index of state anxiety severity. No association was found with the Self-Rating Depression Scale, an index of depression severity. It is reported that rs131702 of *BCR* in Japanese patients are related to bipolar II depression characterized by fluctuation between abnormal mood states of mania and depression. Our results indicate that rs131702 of *BCR* is independent of depression in this study and is, therefore, a prognostic factor unique to tinnitus. We conclude that the severity of tinnitus is associated with genes related to depression.

## Introduction

Tinnitus is a perceived symptom that affects 15% of the population. Although no external sound source can be attributed to tinnitus, in 20% of cases, the patient often experiences severe enough distress, impairing their daily activities^1^. In addition to hearing impairment, tinnitus is associated with different types of clinical symptoms due to the involvement of various pathologies resulting from environmental, psychological (depression or anxiety disorder), or other factors.

In examining tinnitus, the maximum slope within audiograms is determined to be higher in people with tinnitus than in people with hearing loss without tinnitus, despite the latter having a greater mean hearing loss^2^. The additional involvement of non-auditory areas of the brain, particularly areas associated with awareness and salience, can explain why some people with hearing loss develop tinnitus^3,4^. Whether tinnitus is perceived as bothersome or not may be related to the additional involvement of emotion-processing areas^5–7^. Some models have proposed that tinnitus reflects “an emergent property of multiple parallel dynamically changing and partially overlapping sub-networks”. This suggests that various brain networks associated with memory and emotional processing are involved in tinnitus and that the degree of involvement of the different networks reflects the variable aspects of an individual’s tinnitus^3,4,8^.

The psychological models, which use the concept of habituation, explain how and why some people experience a negative effects of tinnitus on quality of life^9^. For example, Jastreboff’s model features classical conditioning mechanisms^10^ and there are also cognitive-behavioral models^11–13^. These models underpin the rationale for and development of cognitive-behavioral interventions for reducing the impact of tinnitus on quality of life.

According to the neurophysiological model of Jastreboff^14^ and the maladaptive neural plasticity model of Shore^15^, tinnitus has been found to be correlated with the plasticity of the central nervous system.

Tinnitus Retraining Therapy (TRT), consisting of sound therapy and psychotherapy based on the neurophysiological model proposed by Jastreboff in the latter half of the 1980s, demonstrates good therapeutic effect as a standard therapy in Japan^16^. Additionally, according to the Multidisciplinary European Guideline for tinnitus^17^ there is evidence for safety but little high-level evidence for the effectiveness of TRT (one RCT and two systematic reviews). However, some tinnitus patients have experienced treatment with difficulties^10^.

There are several tests in use for assessing the level of severity of tinnitus complaints. These questionnaires including Tinnitus Handicap Inventory (THI), Tinnitus Questionnaire, and Visual Analogue Scale have been used to examine tinnitus distress and related factors.

In recent years, gap detection tests, proposed by Turner in 2006^18^, for patients with tinnitus^19^ and tinnitus animal models has enabled the investigation of tinnitus pathology. Gene analysis is also identified as an effective method for elucidating disease pathology and developing novel therapeutic approaches. Thus, in the field of otolaryngology, next-generation sequencing (NGS) can be used to identify the causative genes in congenital deafness, elucidate the pathology, and predict the phenotype of hearing loss. In recent years, there have been reports of tinnitus-related gene analysis being used to clarify the mechanisms of tinnitus and discover tinnitus biomarkers.

Sand et al. analyzed the relevance of tinnitus and single nucleotide polymorphisms (SNPs) in brain-derived neurotrophic factor (*BDNF*) and glial-derived neurotrophic factor (*GDNF*) genes. Some genotypes predicted tinnitus severity in women (but not in men). There was no significant difference in mean tinnitus severity scores between carriers and non-carriers of the minor alleles (p > 0.19), nor did a positive family history of tinnitus in first-degree relatives predict minor allele carrier status (p = 0.08)^20^. There are, however, conflicting reports on the association between tinnitus and plasma or serum BDNF. Goto et al. reported that plasma BDNF levels were significantly higher in the group of patients mildly handicapped by tinnitus than in the severely handicapped and control groups (p < 0.01)^21^. Xiong et al. found plasma BDNF levels were elevated in patients with tinnitus compared with healthy controls^22^. Coskunoglu et al. found lower serum BDNF levels in tinnitus patients than controls. There was no correlation between *BDNF* gene polymorphism and tinnitus^23^.The contribution of BDNF to tinnitus severity is still under debate as mentioned above.

*KCTD12* genes are auxiliary subunits of GABAB receptors, and GABAB receptor agonists are known tinnitus suppressors^24^.

Previous reports proposed that the *KCNE3* gene involved in voltage-gated potassium channels correlated to non-syndromic hearing loss. Implication of the *KCNE3* gene in tinnitus was investigated; however, no *KCNE3* variants was implicated^25^.

Deniz et al. reported *SLC6A4* as a candidate for the serotonin transporter gene^25^. Although no SNPs associated with tinnitus have been identified, Deniz et al. identified a significant association between the 5-HTTLPR polymorphism and scores from the Visual Analogue Scale of the patients. The generation of the tinnitus signal is not associated with *SLC6A4* polymorphism and possibly with serotonergic mechanisms. However, the genotype variant of the *SLC6A4* polymorphic promoter region seems associated with the limbic and autonomic nervous system symptoms of patients with tinnitus. Selective serotonin reuptake inhibitors are also determined to be effective in reducing tinnitus distress, indicating that further elucidation of the relationship between tinnitus and *SLC6A4* should be considered^25^.

*COCH* is a known causative gene of dominantly inherited non-syndromic hearing loss. An association between tinnitus and *COCH* gene mutation has been reported in the analysis of a family with the mutation^26^. The study involved a basic genetic analysis related to tinnitus. They identified a heterozygous 18 base pair deletion on exon 11 of the *COCH* gene in a large multigenerational family, segregating late-onset progressive bilateral sensorineural hearing impairment and tinnitus. However, hearing loss can explain the association between tinnitus and the *COCH* variant, judging from the fact that the tinnitus of patients with *COCH* variants is secondary from hearing loss.

In another pilot study, Gilles et al. performed a genome-wide association study^27^ of 4,000,000 SNPs in 167 patients experiencing tinnitus lasting > 5 min and 749 non-tinnitus groups. However, no clearly related SNPs were detected.

Despite the existence of several reports on the tinnitus-related genes, their contribution to the development of tinnitus is still under debate due to the variety of tinnitus phenotypes caused by clinical heterogeneity such as anxiety, age, hearing loss, occupational/recreational noise exposure, and distress. Among the phenotype variety, psychological factors in the heterogeneous aspects of tinnitus are reported to be most influential to the THI score. Additionally, the psychological models proposed the involvement of memory and emotional processing area in the central nerve system in the tinnitus. Thus, the hypothesis was that the focus on the gene linked with psychological factors such as anxiety, depression, fear and panic disorder can facilitate the exploration of genetically contributing factors to tinnitus distress.

Therefore, we analyzed the relationship between genetic backgrounds correlated to psychological condition and tinnitus distress levels and searched for prognostic markers. Online Mendelian Inheritance in Man (OMIM) lists the genes contributing to depression/anxiety, fear, and panic disorder (https://www.omim.org/). Among them, we found eight SNPs with a minor allele frequency (MAF) more than or equal to 0.1 according to the HapMap for Japan.

Three SNPs (rs140504, rs131690, and rs131702)^28^ of *BCR* in Japanese patients are related to bipolar II depression characterized by fluctuation between abnormal mood states of mania and depression. Bipolar I patients experience full manic episodes, while a person with bipolar II will experience only hypomanic episodes.

The rs1545843 allele (MAF = 0.41 in controls) of Solute Carrier Family6 (Neurotransmitter Transporter), Member 15; *SLC6A15* gene on chr12q21.31 showed experiment-wide significance in a recessive mode of inheritance (AA versus AG+GG) on 12q21.31 and was associated with a higher risk for major depression, but only in individuals less than 55 years old and only when homozygous with an odds ratio of 1.4 (p = 2 × 10e-8).

The rs2267735 allele of Adenylate Cyclase-activating polypeptide1 Receptor For; *ADCYAP1R1* predicts post-traumatic stress disorder (PTSD) diagnoses and symptoms in females only. This may occur due to the estrogen regulation of *ADCYAP1R1*. Total PTSD symptoms are differentially associated with rs2267735 genotype (CC is a high-risk variant) in females (p ≤ 0.001).

The SNP of the serotonin 2A receptor; *HTR2A,* rs7997012, is associated with the outcome of antidepressant treatment^29^.

The rs10997870 allele of Sirtuin 1; *SIRT1* is activates Monoamine oxidase A; *MAO-A* in the brain to mediate anxiety and exploratory drive^30^ and the rs1799836 in Monoamine oxidase B; *MAOB* affect the levels of negative emotions in healthy human volunteers^31^.

## Results

**Figure 1:**
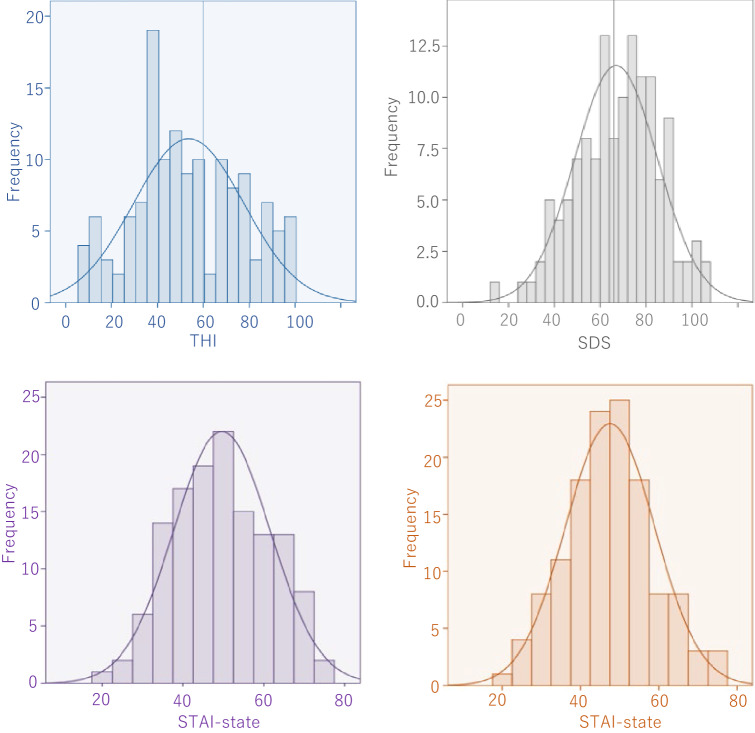
Results of patient questionnaires. Although the THI score was generally biased toward moderate symptoms, the results were globally distributed from mild to severe. The scores of STAI-state, STAI-trait, and SDS characteristics were broadly distributed from mild to severe; it was not a normal distribution. THI, Tinnitus Handicap Inventory; SDS, Self-Rating Depression Scale; STAI, State-Trait Anxiety Inventory.

The average THI score was 53.64 (s.d. 24.05). The average Self-Rating Depression Scale (SDS) score was determined to be at 43.48 (s.d. 9.05). The average STAI-state score was 49.61 (s.d. 11.97). The average STAI-trait score was 47.51 (s.d. 11.41). The THI score, the SDS score, the STAI-state score, and the STAI trait score were distributed broadly from mild to severe, although they did not exhibit a normal distribution (Fig. 1, Table 1).

**Table 1:** Clinical information of the subjects.

	Total			Male			Female		
	N	Mean	SD	N	Mean	SD	N	Mean	SD
Age	138	61.31	13.12	59	60.92	13.21	79	61.61	13.13
THI	138	53.64	24.05	59	54.58	21.72	79	52.95	25.77
SDS	131	43.48	9.05	56	44.91	8.68	75	42.41	9.23
STAI-State	132	49.61	11.97	55	51.82	10.66	77	48.03	12.65
STAI-Trait	131	47.51	11.4	54	48.81	10.45	77	46.6	12.01

The association between SNP rs131702 of the BCR gene and the severity of THI was found to be significant (Tables 2, 3). The results of the Fisher test indicated that the carriers of minor allele G showed a severe THI score with odds ratio of 2.019 (Table 2). The trend test also revealed that the minor allele G was positively associated with the severity of the THI score (Table 3). Furthermore, there was a significant allelic association between SNP rs131702 of the BCR gene and the STAI-state score with the trend test. The severe STAI-state score, as well as the severe THI score was detected in the carriers of the minor allele G (Table 4). On the other hand, SNP rs131702 did not show any association with the STAI-trait score and the SDS (Tables 5, 6). No other SNP showed any relation with the THI, STAI, or SDS score.

**Table 2:** Statistical analysis for single SNP; the comparison of allele frequencies between cases with mild-moderate THI and with severe-catstrophic THI.

Gene	SNP	Allele	All subjects (n = 134)				
			Mild and moderate (%)	Severe and catastrophic (%)	Total	*P for Fisher's test*	Odds ratio (for considering minor allele as risk allele)
*BCR*	rs140504	A allele	92(57.5)	49(45.4)	141(52.6)	0.061	1.629 (0.996–2.664)
		G allele	68(42.5)	59(54.6)	127(47.4)		
		Total	160	108	268		
*BCR*	rs131690	A allele	123(76.9)	79(73.1)	202(75.4)	0.563	1.22 (0.695–2.141)
		G allele	37(23.1)	29(26.9)	66(24.6)		
		Total	160	108	268		
*BCR*	rs131702	G allele	53(33.1)	53(50)	106(39.8)	0.007	2.019 (1.220–3.340)
		T allele	107(66.9)	53(50)	160(60.2)		
		Total	160	106	266		
*SLC6A15*	rs1545843	A allele	53(33.1)	41(38)	94(35.1)	0.436	1.235 (0.742–2.056)
		G allele	107(66.9)	67(62)	174(67.9)		
		Total	160	108	268		
*ADCYAP1R1*	rs2267735	C allele	81(50.6)	48(44.4)	129(48.1)	0.383	0.78 (0.478–1.274)
		G allele	79(49.4)	60(55.6)	139(51.9)		
		Total	160	108	268		
*SIRT1*	rs10997870	G allele	131(81.9)	87(80.6)	218(81.3)	0.873	1.353 (0.659–2.780)
		T allele	29(18.1)	21(19.4)	50(18.7)		
		Total	160	108	268		
*HTR2A*	rs7997012	A allele	32(20)	25(23.1)	57(21.3)	0.546	1.205 (0.667–2.177)
		G allele	128(80)	83(76.9)	211(78.7)		
		Total	160	108	268		
*MAOB (male)*	rs1799836	C allele	4(12.1)	3(13)	7(12.5)	1	1.088 (0.219–5.395)
		T allele	29(87.9)	20(87)	49(87.5)		
		Total	33	23	56		
*MAOB (female)*	rs1799836	C allele	19(22.1)	8(13.3)	27(18.5)	0.201	0.543 (0.220–1.337)
		T allele	67(77.9)	52(86.7)	119(81.5)		
		Total	86	60	146

**Table 3:** Statistical analysis for single SNP to evaluate the correlation between genotype and THI score.

Gene	SNP	Genotype	All Subjects (n =134)			
			N	Mean	SD	*P for trend*
*BCR*	rs140504	AA	35	47.26	22.57	0.103
		AG	71	55	23.75	
		GG	28	56.86	26.22	
*BCR*	rs131690	AA	76	52.72	24.8	0.567
		AG	50	53.36	22.51	
		GG	8	59.5	28.96	
*BCR*	rs131702	GG	18	66.67	24.49	0.008
		GT	70	53.3	24.04	
		TT	45	47.82	22.53	
*SLC6A15*	rs1545843	AA	17	50	13.95	0.514
		AG	60	57.33	25.8	
		GG	57	50.19	24.31	
*ADCYAP1R1*	rs2267735	CC	29	53.55	25.12	0.763
		CG	71	52.39	24.21	
		GG	34	55.24	23.55	
*SIRT1*	rs10997870	GG	90	53.78	25.78	0.772
		GT	38	52.63	20.46	
		TT	6	51.83	22.26	
*HTR2A*	rs7997012	AA	9	60	30.74	0.308
		AG	39	54.82	23.44	
		GG	86	52.01	23.79	
*MAOB (male)*	rs1799836	C	7	65.43	22.11	0.195
		T	49	53.96	21.59	
*MAOB (female)*	rs1799836	CC	2	51	60.81	0.343
		CT	23	47.04	23.64	
		TT	48	54.19	25.86

**Table 4:** Statistical analysis for single SNP to evaluate the correlation between the genotypes and STAI-state score.

Gene	SNP	Genotype	All Subjects (n = 128)			
			N	Mean	SD	*P* for trend
*BCR*	rs140504	AA	35	46.89	11.85	0.101
		AG	65	50.2	11	
		GG	28	51.75	13.79	
*BCR*	rs131690	AA	73	48.68	11.46	0.193
		AG	47	50.28	12.63	
		GG	8	54.5	11.92	
*BCR*	rs131702	GG	18	54.56	13.31	0.016
		GT	66	50.15	11.62	
		TT	43	46.67	11.37	
*SLC6A15*	rs1545843	AA	15	45.4	8.89	0.908
		AG	58	52	12.27	
		GG	55	48.29	11.92	
*ADCYAP1R1*	rs2267735	CC	28	47.57	13.2	0.146
		CG	70	49.39	11.96	
		GG	30	52.13	0.45	
*SIRT1*	rs10997870	GG	85	50.22	12.33	0.897
		GT	37	47.19	10.74	
		TT	6	56.33	11.15	
*HTR2A*	rs7997012	AA	9	44.78	12.68	0.895
		AG	39	51.59	13.03	
		GG	80	49.23	11.22	
*MAOB* (male)	rs1799836	C	6	55.33	13.87	0.478
		T	46	52	10.33	
*MAOB* (female)	rs1799836	CC	2	60	19.8	0.35
		CT	23	47.7	12.94	
		TT	46	46.91	12.04

**Table 5:** Statistical analysis for single SNP to evaluate the correlation between the genotypes and STAI-trait score.

Gene	SNP	Genotype	All subjects (n = 127)			
			N	Mean	SD	*P for trend*
*BCR*	rs140504	AA	35	45.91	10.6	0.331
		AG	65	47.77	10.76	
		GG	27	48.67	13.34	
*BCR*	rs131690	AA	73	46.75	10.66	0.127
		AG	46	47.09	11.83	
		GG	8	55.88	11.31	
*BCR*	rs131702	GG	18	50.72	11.85	0.076
		GT	65	47.78	11.61	
		TT	43	45.28	10.24	
*SLC6A15*	rs1545843	AA	14	45.07	10.87	0.31
		AG	58	50.05	11.31	
		GG	55	45.31	10.91	
*ADCYAP1R1*	rs2267735	CC	28	46.71	11.67	0.211
		CG	70	46.51	11.61	
		GG	29	50.41	9.78	
*SIRT1*	rs10997870	GG	84	47.29	12.01	0.342
		GT	37	46.32	9.28	
		TT	6	56.67	8.5	
*HTR2A*	rs7997012	AA	9	47.33	11.74	0.536
		AG	39	48.77	13.23	
		GG	79	46.81	10.21	
*MAOB (male)*	rs1799836	C	6	54.17	16.27	0.231
		T	45	48.62	9.65	
*MAOB (female)*	rs1799836	CC	2	50.5	13.44	0.521
		CT	23	46.74	10.7	
		TT	46	45.46	12.26

**Table 6:** Statistical analysis for single SNP to evaluate the correlation between the genotypes and SDS score.

Gene	SNP	Genotype	All subjects (n = 127)			
			N	Mean	SD	*P for trend*
*BCR*	rs140504	AA	35	41.63	8.71	0.218
		AG	64	43.8	8.36	
		GG	28	44.36	10.7	
*BCR*	rs131690	AA	73	43.4	8.7	0.899
		AG	46	42.91	9.54	
		GG	8	45	9.55	
*BCR*	rs131702	GG	18	45.11	9.71	0.169
		GT	64	43.7	9.24	
		TT	44	41.89	8.41	
*SLC6A15*	rs1545843	AA	15	42.53	9.4	0.381
		AG	58	44.76	8.98	
		GG	54	42	8.86	
*ADCYAP1R1*	rs2267735	CC	27	42.59	9.06	0.229
		CG	70	42.71	8.9	
		GG	30	45.4	9.18	
*SIRT1*	rs10997870	GG	85	43.34	9.28	0.537
		GT	37	42.3	8.44	
		TT	5	50.6	5.32	
*HTR2A*	rs7997012	AA	9	39.44	9.29	0.666
		AG	39	44.41	10.2	
		GG	79	43.23	8.31	
*MAOB (male)*	rs1799836	C	7	47.71	9.86	0.453
		T	46	45.07	8.46	
*MAOB (female)*	rs1799836	CC	2	47.5	9.19	0.203
		CT	23	43.13	8.29	
		TT	44	40.86	9.52	

Multiple regression analysis was performed to assess whether the STAI-state score was a confounder between the THI score and SNP rs131702 of the BCR gene (Table 7). The THI score is coded as 1 = 56 and under and 2 = 58 and over. STAI-state is coded as 1 = 44 or under for male, 40 or under for female and 2 = 45 and over for male, 41 and over for female. The genotype of SNP rs131702 of BCR gene is coded as 0 = TT, 1 = GT, 2 = GG. The THI score was identified as the dependent variable and STAI-state and SNP rs131702 as independent variables. The regression coefficient associated with the rs131702 of BCR was 0.175 in the simple linear regression analysis. The association was statistically significant (p = 0.008). The association between THI score and SNP rs131702 of BCR was 0.147 after adjustment for the STAI-state score. SNP rs131702 of BCR retains a statistically significant association with THI score (p = 0.02), but the value of the regression coefficient decreased by 16.7%.

**Table 7:** (a) The simple linear regression analysis for THI score and (b) the multiple regression analysis for THI score.

	Regression coefficient	P value
**(a) Independent variables**		
SNP rs131702 of *BCR* gene	0.175566826	0.008047161
STAI-state	0.317073171	0.000621627
**(b) Independent variables**		
SNP rs131702 of *BCR* gene	0.147354015	0.021741808
STAI-state	0.288321168	0.001639804

## Discussion

We theorized there might be an association between the genetic background related to psychological factors and the grade of tinnitus distress, which might lead us to identify prognostic markers.

The *BCR* SNP rs131702 is found on chromosome 22. The *BCR* gene encodes the Rho family low molecular weight G-protein, which is abundantly expressed in the central nervous system and considered crucial for neurogenesis.

The results could support the suggestion that the *BCR* gene is implicated in the process of making tinnitus distress severe. The SDS score and the STAI-state score are known to contribute significantly to the THI score^14,32^. Therefore, in addition to the THI score, we analyzed the association between these two scores and each SNPs.

In 171 Japanese patients, bipolar depression and related disorders (odds ratio minor allele (G) 1.49, CI: 1.15–1.93, *p* = 0.0026) have been reported to be associated with rs131702 (https://www.snpedia.com/index.php/Rs131702).

In this study, no significant association between *BCR* rs131702 and the SDS score was detected, because the patients with mental illness were excluded.

In this study, a significant association between rs131702 of *BCR* and the severe THI score was recognized. rs131702 was independent of the SDS score and the STAI-trait score. But a significant association between rs131702 and the severe STAI-state score was identified. It could be considered that the STAI-state score affected the result of the correlation between the severe THI score and rs131702 as a confounding factor, as the STAI-state score has a significant association with both the THI score and rs131702. Therefore, multiple regression analysis was then performed to assess whether the STAI-state score is a confounder. Although the association between the severe THI score and rs131702 was significant even after adjustment for the STAI-state score, the value of the coefficient after adjustment decreased by about 16%. On the other hand, the coefficient of association between THI score and STAI-state score was almost the same even after adjustment for rs131702. It may indicate that the part of the association between the severe THI score and rs131702 of *BCR* can be explained by the STAI-state score, because the change by more than 10% of the regression coefficient in the multiple linear regression model indicates the influence of cofounders.

Although the STAI-state score was identified as cofounder between THI score and SNP rs131702 of the *BCR* gene, it could mean that the patients with SNP rs131702 of *BCR* gene have potential to suffer from more severe tinnitus. Therefore, it is considered a prognostic factor specific to tinnitus.

The study had several limitations. Because of the small sample size, our ability to evaluate other patient groups to confirm reproducibility was limited. Further, we evaluated only eight candidate genes. The likelihood of tinnitus being caused by multiple genes (susceptibility genes) is high. Therefore, future studies should examine more candidate genes. Finally, we limited our study to those with a minor allele frequency ≥ 0.1, but this could have eliminated a strongly associated SNP or a rare variant. Therefore, the sample size and number of genes evaluated would need to be increased in future studies.

## Subjects and methods

This study was approved by the Ethics Committee of Keio University (UMIN000017306). The research was performed in accordance with clinical research guidelines in Japan, and informed consent was obtained from all patients.

### Subjects

We enrolled patients who presented to the Department of Otolaryngology, Keio University Hospital, Japan, from January 2013 until April 2015, who complained of tinnitus continuing for over 3 months (the average duration was 56.2 ± 82.0 months**)** which is defined as chronic tinnitus in Japan^16^. After excluding patients with mental illness, a final cohort of 138 patients remained. These patients answered the validated Japanese version of THI^33,34^, SDS^35,36^, and STAI questionnaires^37,38^, and blood samples were drawn to investigate SNPs.

### Genotyping

Genomic DNA samples were extracted from the peripheral blood of patients using a DNA extraction kit (Genomix, Biologica, Japan).

Genotyping of the eight SNPs (rs140504^28^, rs131690^28^, rs131702^28^, rs1545843^39^, rs2267735^40^, rs10997870^30^, rs7997012^29^, and rs1799836^31^) was performed by allelic discrimination analysis (Light cycler 480 system,Roche, Basel, Switzerland), using TaqMan SNP genotyping assay probes (Thermo Fisher Scientific, Waltham, USA. The genotyping call rate was 100%.

The correlation between a genetic background related to depression/anxiety and tinnitus distress levels was evaluated. The SNPs targeted in our study were determined to be correlated with depression, anxiety disorder, and obsessive–compulsive disorder, according to Online Mendelian Inheritance in Man (https://www.omim.org/). We found eight SNPs with a MAF more than or equal to 0.1 according to the HapMap for Japan (Table 8).

**Table 8:** Candidate gene polymorphism.

Gene	Chromosome	Polymorphism	Genotypes	Reported minor allele	Reported minor allele frequency (JPT)
*BCR*	22	rs140504	GG/GA/AA	G	0.500*
*BCR*	22	rs131690	GG/GA/AA	G	0.235*
*BCR*	22	rs131702	GG/GA/AA	G	0.407*
*SLC6A15*	12	rs1545843	AA/AG/GG	A	0.367*
*ADCYAP1R1*	7	rs2267735	GG/GC/CC	C	0.479*
*SIRT1*	10	rs10997870	TT/TG/GG	T	0.149*
*HTR2A*	13	rs7997012	AA/AG/GG	A	0.173^+^
*MAOB* (male)	X	rs1799836	C/T	C	0.147^#^
*MAOB* (female)		rs1799836	CC/CT/TT		

*Integrative Japanese Genome Variation Database, ^+^1000 Genomes: phase_3 (JPT), ^#^Hapmap_JPT.

The target SNPs were analyzed using the TaqMan method.

In this study, a THI score of less than 56 was considered light/moderate, and a score greater than or equal to 58 was considered severe^33^. Both trait anxiety (STAI-trait) and state anxiety (STAI-state) are evaluated in the STAI. High trait anxiety implies a remarkable tendency to respond with anxiety to perceived threats in the environment, and high state anxiety implies a transitory high symptom of anxiety. The depression level is measured by the SDS. Higher score of the SDS indicates a severer depressive condition. Statistical analysis was performed using Fisher’s exact test (software PLINK v1.07 https://zzz.bwh.harvard.edu/plink/index.shtml).

Furthermore, the association between SNPs and the severity of THI, SDS, and STAI was evaluated using the Jonckheere trend test. In each of the above tests, the significance level was set at 5%.

## References

[1] Tunkel DE (2014). Clinical practice guideline: Tinnitus executive summary. Otolaryngol. Head Neck Surg..

[2] Konig O, Schaette R, Kempter R, Gross M (2006). Course of hearing loss and occurrence of tinnitus. Hear. Res..

[3] De Ridder D, Elgoyhen AB, Romo R, Langguth B (2011). Phantom percepts: Tinnitus and pain as persisting aversive memory networks. Proc. Natl. Acad. Sci. USA.

[4] De Ridder D (2014). An integrative model of auditory phantom perception: Tinnitus as a unified percept of interacting separable subnetworks. Neurosci. Biobehav. Rev..

[5] Rauschecker JP, Leaver AM, Muhlau M (2010). Tuning out the noise: Limbic–auditory interactions in tinnitus. Neuron.

[6] Schecklmann M (2013). Neural correlates of tinnitus duration and distress: A positron emission tomography study. Hum. Brain Mapp..

[7] Vanneste S, Joos K, De Ridder D (2012). Prefrontal cortex based sex differences in tinnitus perception: Same tinnitus intensity, same tinnitus distress, different mood. PLoS One.

[8] Elgoyhen AB, Langguth B, De Ridder D, Vanneste S (2015). Tinnitus: Perspectives from human neuroimaging. Nat. Rev. Neurosci..

[9] Hallam, R. Psychological aspects of tinnitus*. In Contributions to Medical Psychology*. Vol. 84 (Oxford, 1984).

[10] Jastreboff PJ (1990). Phantom auditory perception (tinnitus): Mechanisms of generation and perception. Neurosci. Res..

[11] McKenna L, Handscomb L, Hoare DJ, Hall DA (2014). A scientific cognitive-behavioral model of tinnitus: Novel conceptualizations of tinnitus distress. Front. Neurol..

[12] Cima RF, Crombez G, Vlaeyen JW (2011). Catastrophizing and fear of tinnitus predict quality of life in patients with chronic tinnitus. Ear Hear..

[13] Kleinstauber M (2013). The role of fear-avoidance cognitions and behaviors in patients with chronic tinnitus. Cogn. Behav. Ther..

[14] Jastreboff PJ, Hazell JW (1993). A neurophysiological approach to tinnitus: Clinical implications. Br. J. Audiol..

[15] Shore SE, Roberts LE, Langguth B (2016). Maladaptive plasticity in tinnitus–triggers, mechanisms and treatment. Nat. Rev. Neurol..

[16] Ogawa K (2019). Clinical practice guidelines for diagnosis and treatment of chronic tinnitus in Japan. Auris Nasus Larynx.

[17] Cima RFF (2019). A multidisciplinary European guideline for tinnitus: Diagnostics, assessment, and treatment. Hno.

[18] Turner JG (2006). Gap detection deficits in rats with tinnitus: A potential novel screening tool. Behav. Neurosci..

[19] Boyen K, Baskent D, van Dijk P (2015). The gap detection test: Can it be used to diagnose tinnitus?. Ear Hear..

[20] Sand PG, Langguth PG, Schecklmann M, Kleinjung T (2012). GDNF and BDNF gene interplay in chronic tinnitus. Int. J. Mol. Epidemiol. Genet..

[21] Goto F (2012). Various levels of plasma brain-derived neurotrophic factor in patients with tinnitus. Neurosci. Lett..

[22] Xiong H (2016). Plasma brain-derived neurotrophic factor levels are increased in patients with tinnitus and correlated with therapeutic effects. Neurosci. Lett..

[23] Coskunoglu A (2017). Evidence of associations between brain-derived neurotrophic factor (BDNF) serum levels and gene polymorphisms with tinnitus. Noise Health.

[24] Sand PG (2012). Resequencing of the auxiliary GABA(B) receptor subunit gene KCTD12 in chronic tinnitus. Front. Syst. Neurosci..

[25] Deniz M (2010). Significance of serotonin transporter gene polymorphism in tinnitus. Otol. Neurotol..

[26] Gallant E (2013). Novel COCH mutation in a family with autosomal dominant late onset sensorineural hearing impairment and tinnitus. Am. J. Otolaryngol..

[27] Gilles A, Van Camp G, Van de Heyning P, Fransen E (2017). A pilot genome-wide association study identifies potential metabolic pathways involved in tinnitus. Front. Neurosci..

[28] Hashimoto R (2005). The breakpoint cluster region gene on chromosome 22q11 is associated with bipolar disorder. Biol. Psychiatry.

[29] McMahon FJ (2006). Variation in the gene encoding the serotonin 2A receptor is associated with outcome of antidepressant treatment. Am. J. Hum. Genet..

[30] Libert S (2011). SIRT1 activates MAO-A in the brain to mediate anxiety and exploratory drive. Cell.

[31] Dlugos AM, Palmer AA, de Wit H (2009). Negative emotionality: Monoamine oxidase B gene variants modulate personality traits in healthy humans. J. Neural. Transm. (Vienna).

[32] Oishi N (2011). Influence of depressive symptoms, state anxiety, and pure-tone thresholds on the tinnitus handicap inventory in Japan. Int. J. Audiol..

[33] Newman CW, Jacobson GP, Spitzer JB (1996). Development of the Tinnitus Handicap Inventory. Arch. Otolaryngol. Head Neck Surg..

[34] Shinden S, Ogawa K, Inoue Y, Tazoe M, Asano K (2002). Methods for assessing the level of distress and difficulty in activities of daily living due to tinnitus. Audiol. Jpn..

[35] Zung W (1965). A self-rating depression scale. Arch. Gen. Psychiatry.

[36] Zung, W., Fukuda, K., Kobayashi, S. & Shiyoutebiki, SDS. (Sankyobo, Kyoto, 1983).

[37] Spielberger C (1984). State-Trait Anxiety Inventory: A Comprehensive Bibliography.

[38] Hidano T, Fukuhara M, Iwawaki M, Soga S, Spielberger C (2000). State-Trait Anxiety Inventory-JYZ.

[39] Kohli MA (2011). The neuronal transporter gene SLC6A15 confers risk to major depression. Neuron.

[40] Ressler KJ (2011). Post-traumatic stress disorder is associated with PACAP and the PAC1 receptor. Nature.

